# Audio-biofeedback versus the scale method for improving partial weight-bearing adherence in healthy older adults: a randomised trial

**DOI:** 10.1007/s00068-024-02609-5

**Published:** 2024-08-17

**Authors:** Arlene Vivienne von Aesch, Sonja Häckel, Tobias Kämpf, Heiner Baur, Johannes Dominik Bastian

**Affiliations:** 1 Physiotherapie SportClinic Zurich, Giesshübelstrasse 15, 8045 Zurich, Switzerland; 2 Department of Orthopaedic Surgery and Traumatology, Inselspital, University Hospital Bern, University of Bern, Bern, Switzerland; 3 School of Health Professions, Department of Physiotherapy, Bern University of Applied Sciences, Murtenstrasse 10, Bern, Switzerland

**Keywords:** Randomized controlled trial, Aged, Weight-bearing, Feedback, Sensory, Weights and measures, Rehabilitation

## Abstract

**Purpose:**

To investigate how audio-biofeedback during the instruction of partial weight-bearing affected adherence, compared to traditional methods, in older adults; and to investigate the influence of individual characteristics.

**Methods:**

The primary outcome measure of this randomised controlled trial was the amount of load, measured as the ground reaction force, on the partial weight-bearing leg. The secondary outcome was the influence of individual characteristics on the amount of load. Included were healthy volunteers 60 years of age or older without gait impairment. Participants were randomly allocated to one of two groups; blinding was not possible. Partial weight-bearing of 20 kg was trained using crutches with audio-biofeedback (intervention group) or a bathroom scale (control group). The degree of weight-bearing was measured during six activities with sensor insoles. A mean load between 15 and 25 kg was defined as adherent.

**Results:**

There was no statistically significant difference in weight-bearing between the groups for all activities measured. For the sit-stand-sit activity, weight-bearing was within the adherence range of 15–25 kg (audio-biofeedback: 21.7 ± 16.6 kg; scale: 22.6 ± 13 kg). For standing, loading was below the lower threshold (10 ± 7 vs. 10 ± 10 kg). Weight-bearing was above the upper threshold for both groups for: walking (26 ± 11 vs. 34 ± 16), step-up (29 ± 18 vs. 34 ± 20 kg) and step-down (28 ± 15 vs. 35 ± 19 kg). Lower level of cognitive function, older age, and higher body mass index were correlated with overloading.

**Conclusion:**

Audio-biofeedback delivered no statistically significant benefit over the scale method. Lower cognitive function, older age and higher body mass index were associated with overloading.

**Trial registration:**

Not applicable due not being a clinical trial and due to the cross-sectional design (one measurement point, no health intervention, no change in health of a person).

## Introduction

A period of partial weight-bearing (PWB) is often prescribed during rehabilitation of fractures and after orthopaedic surgery of the lower limb [1, 2]. Reducing weight-bearing decreases the risk of fracture malunion and displacement [3, 4], while mobilisation with some load supports bone healing and improves outcomes after surgery [5, 6]. Because there is no standardised recommendation, the amount, duration, and instruction of PWB vary depending on the diagnosis, treating clinician, and hospital [7–9].

The analogue bathroom scale is considered the gold standard instrument for learning a PWB load. However, verbal instruction, sometimes combined with tactile methods and demonstration, is the most commonly used method [10, 11]. Many studies have shown that participants do not consistently meet weight-bearing targets after any type of traditional PWB instruction [10, 12, 13]. The reason for this poor adherence remains unclear, but is likely to be multifactorial. One theory is that patients may have difficulty gauging and controlling the degree of weight-bearing due to influencing factors, such as pain or inadequate strength [14, 15]. Another theory is that the scale is best suited to measuring load in a static situation and is therefore inappropriate for learning PWB in a dynamic activity such as walking.

Because poor adherence to PWB could compromise recovery, the study of alternative training methods is essential. Improvement is especially important for patients over the age of 60, as the incidence of lower limb fractures and orthopaedic surgeries is high in this population group [16], places a significant burden on healthcare systems [16], and older age correlates highly with poorer PWB adherence [15, 17]. Fortunately, technological advances have resulted in devices that could improve the monitoring and instruction of weight-bearing during rehabilitation. Sensor insoles in shoes are particularly promising, as they can measure load and provide biofeedback in “real-time” during dynamic activities [18].

This study aimed to determine whether a sensor insole system with audio-biofeedback could improve adherence to PWB in older adults. The primary objective was to estimate the effect of audio-biofeedback versus the scale method on the ability of persons aged 60 years or older to adhere to a PWB target of 20 kg during functional mobility activities. The secondary objective was to investigate the influence of individual characteristics, such as strength, on adherence ability.

## Methods

### Study design and participants

This study was designed as a randomised controlled trial with two independent groups and an allocation ratio of one to one. The responsible federal ethics committee Kantonale Ethikkommission Bern stated, after reviewing, that the study did not fall under the Swiss Human Research Act and did not require further approval (BASEC-Nr.: Req-2021-00554). Trial registration was not applicable due to the cross-sectional design (one measurement point, no intervention).

An a priori sample size was calculated for two independent groups (significance level = 0.05; power = 0.80) and resulted in N = 9 per group [19]. The effect size was based on the results of a similar study with the used mean difference as follows: intervention group mean ± standard deviation (SD) 199 ± 39 N, control group mean 251 N [20]. Participants were recruited by hanging up flyers and sending emails to organisations and healthcare clinics within the local community. Inclusion criteria were being a minimum of 60 years of age, having a European shoe size of 36–45 and being able to navigate stairs and be able to walk without a walking aid for 10 min. Exclusion criteria were the presence of any health issue impacting their gait or ability to use crutches, severe cognitive impairment, and having used crutches within the last six months.

### Data collection

Measurements took place from November 2021 to March 2022 in the Bern Movement Lab at the Bern University of Applied Sciences or in the  SportClinic facilities in Zürich, Switzerland. Volunteers were informed of the study procedures and, if they provided written consent, demographic data were recorded. Grip strength was measured as a quick, valid measure of upper body strength using a hydraulic handgrip dynamometer (Jamar^1^) following the Southampton protocol for adult grip strength measurement [21, 22]. Because cognitive impairment may contribute to poorer compliance in older adults [15, 23], level of cognitive function was evaluated using the German version of the Montreal Cognitive Assessment (MoCA). This validated test is best suited for quickly detecting mild cognitive impairment among people older than 60 years [24, 25]. Levels were defined according to test developers’ recommendations, with no cognitive impairment a score of > 25, mild a score of 18–25, moderate a score of 17–10, and severe a score of < 10 [26].

The primary outcome measure was the amount of weight-bearing on the PWB leg during six functional mobility activities, measured as the ground reaction force in Newtons and converted to kilograms to improve clinical interpretation. Sensor insoles were used to record the ground reaction force (OpenGo, Insole3^2^). The third generation insole3® model is a valid and reliable instrument for measuring vertical ground reaction forces during walking, according to validation studies independent of the producer [27, 28].

### Protocol

Sensor insoles were fitted to the participant and calibrated. The non- (and later partial-) weight-bearing leg was randomly determined using a mobile coin toss application (Tiny Decisions App, Version 2.9.1.^3^). Elbow crutches were fitted and a swing-through non-weight-bearing gait instructed, with the participant given as much time as they needed to feel comfortable and safe [29]. Next, the volunteer was allocated to either the intervention protocol or the control protocol using an online randomisation service, that randomly assigned the individual participant to one of the two groups (Sealed Envelope^4^). Blinding of intervention allocation was not possible. The PWB training protocol was based on the authors’ knowledge of clinical practice and the methodology of similar studies [12, 30–32]. The same experienced and licenced physiotherapist instructed all participants to PWB to a limit of 20 kg according to the protocol of the group to which they were allocated to. No changes were made to the eligibility criteria nor the protocol after commencement of the trial.

In the control group, the target PWB load was instructed using an analogue scale (ADE M308800^5^). The participant stood with their PWB leg on the scale and transferred weight onto this leg until the needle pointed to 20 kg on the dial. They were instructed to pause a moment and try to remember how this degree of load felt before lifting the foot off the scale. They repeated this process five times. Next, the participant stood with the same leg on the scale but looked straight ahead. They were instructed to transfer weight onto this leg until they felt they were loading it to 20 kg, then to look at the dial and correct the pressure, if necessary, before removing the foot off the scale. They repeated this process a total of five times.

For the intervention group, the OpenGo system was used to provide audio-biofeedback using the OpenGo App installed on a smartphone. Using the mobile application’s inbuilt function, 200 N (approximately 20 kg) was manually set as the threshold load for the PWB leg. The participant was informed that, when this function was activated, the application would notify them with a beep when the threshold load of 20 kg had been reached and would continue to beep as long as the load was at or above this threshold. The participant was instructed to place their PWB foot on the floor, transfer weight onto the leg until they heard a beep. Then, they were told to adjust the pressure and, by listening to the biofeedback, learn what amount of pressure constituted 20 kg of load, before lifting their foot off the floor again. The participant repeated this procedure 10 times.

Following, the participant was instructed to walk with a three-point PWB gait and told to try to weight-bear as close as possible too, but not over, 20 kg. They were instructed how to use crutches to stand up from and sit down on a chair and step up onto and step down from a step. In the intervention group, the audio-biofeedback remained activated during this part of training, meaning they had concurrent auditory feedback set to 200 N, whereas the scale group did not. Upon completion of the training session, the audio-biofeedback function was turned off and no further feedback on the amount of weight-bearing was provided to participants in either group. Participants were allowed to practice these activities until they and the physiotherapist were confident that they could perform the activities safely. The training time required varied from approximately 10 to 20 min.

The amount of weight-bearing was recorded at 100 Hz during six functional mobility activities:

1. standing for 30 s,
2. standing up from, and sitting back down on, a chair,
3. stepping up onto a single step,
4. stepping down from a single step,
5. walking on a flat surface for three minutes, and
6. walking with a 4 kg weighted backpack for one minute.

The sit-stand-sit and step activities were measured three times. Prior to each activity, the participant was reminded they should weight-bear close to, but not over, 20 kg.

### Data processing and analysis

The recorded de-identified data was transmitted to the OpenGo Desktop Software (Version 2.1 [see also 2]) and processed using the inbuilt analysis functions. Recordings were deleted from the insole memory immediately after transfer. For the walking activities, the mean of all maxima of total force during the stance phase was reported. For the sit-stand-sit and stepping activities, the maximum total ground reaction force was recorded. For standing, the mean total force during the first 30 s of the recording was used instead, because standing is considered a static activity where the load over time is more relevant than the maximum load. All demographic and sensor insole data were entered into an Excel® spreadsheet on a password-protected computer and hardcopies stored in a locked cabinet.

Statistical analyses were performed using R (Version 4.1.2^6^). Means and standard deviation of PWB were calculated for each group for all activities and because data were not normally distributed (as tested by the Shapiro–Wilk Test) the Mann–Whitney U Test was used to compare the differences between the two groups. Individual and group adherence to weight-bearing was evaluated by comparing PWB means against the 20 kg target load within a pre-defined 10 kg buffer zone (upper limit: 25 kg, lower limit: 15 kg). The selected adherence range was comparable to those used by similar studies [33, 34]. For each participant, the number of steps within and outside this adherence range was manually counted and step percentages for each load classification (adherent, non-adherent overload and non-adherent underload) calculated. The relationship between each independent factor (age, gender, body mass index (BMI), grip strength and MoCA score) and the dependent variable mean PWB load (kg), was assessed via simple linear regression. Finally, multiple linear regression analysis was performed using background knowledge to guide the development of three different models, for each model, the Akaike information criterion was calculated to select the best-fit model across all activities [35]. The confidence interval (CI) was set at 95% and a p-value of 0.05 was defined as statistically significant.

## Results

### Participants

**Fig. 1:**
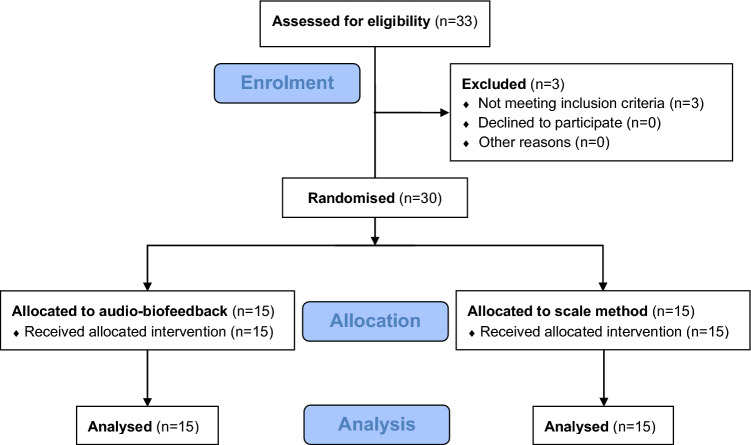
Flow diagram of participants from enrolment to analysis

Participant enrolment began in October 2022 and was completed in February 2022. Of the 33 volunteers, three did not meet eligibility criteria due to having used crutches within the last six months (n = 2) and European shoesize < 36 (n = 1). There were no drop-outs and the trial was stopped after 30 participants had been measured (Fig. 1). There were no harms or accidents during the trial.

Of the thirty included volunteers (16 females and 14 males, age 70.9 ± 6.0 years) 15 were randomly allocated to the intervention group and 15 to the control group. The baseline characteristics between the audio-biofeedback and control group were similar (Table 1). Overall, participants were slightly overweight with normal age-related grip strength and without cognitive impairment.

**Table 1:** Characteristics of the audio-biofeedback and the control groups

Characteristic	Audio (n = 15)	Scale (n = 15)
Sex (female/male)	7/8	9/6
Age (years)	70 ± 5.2	71.8 ± 6.8
Weight (kg)	73.7 ± 18.6	74.9 ± 15.1
BMI (kg/m2)*	25.7 ± 5.1	25.9 ± 4.7
Grip strength (kg)	36.3 ± 7.6	35.3 ± 14
MoCA Score^†^	27.1 ± 2.3	25.9 ± 2.6
Retired (no/yes)	2/13	4/11
PWB leg (left/right)	7/8	7/8
Shoesize (European)	40.7 ± 2.3	41 ﻿± 2.8

Values are expressed as mean ± standard deviation, or as otherwise indicated

*BMI* body mass index, *MoCA* Montreal Cognitive Assessment, *PWB* partial weight-bearing

*Underweight < 18.5, normal 18.5–25, overweight 25–30, obese > 30

^†^No cognitive impairment > 25, mild cognitive impairment 18–25, moderate 17–10, severe < 10

### Outcomes

**Fig. 2:**
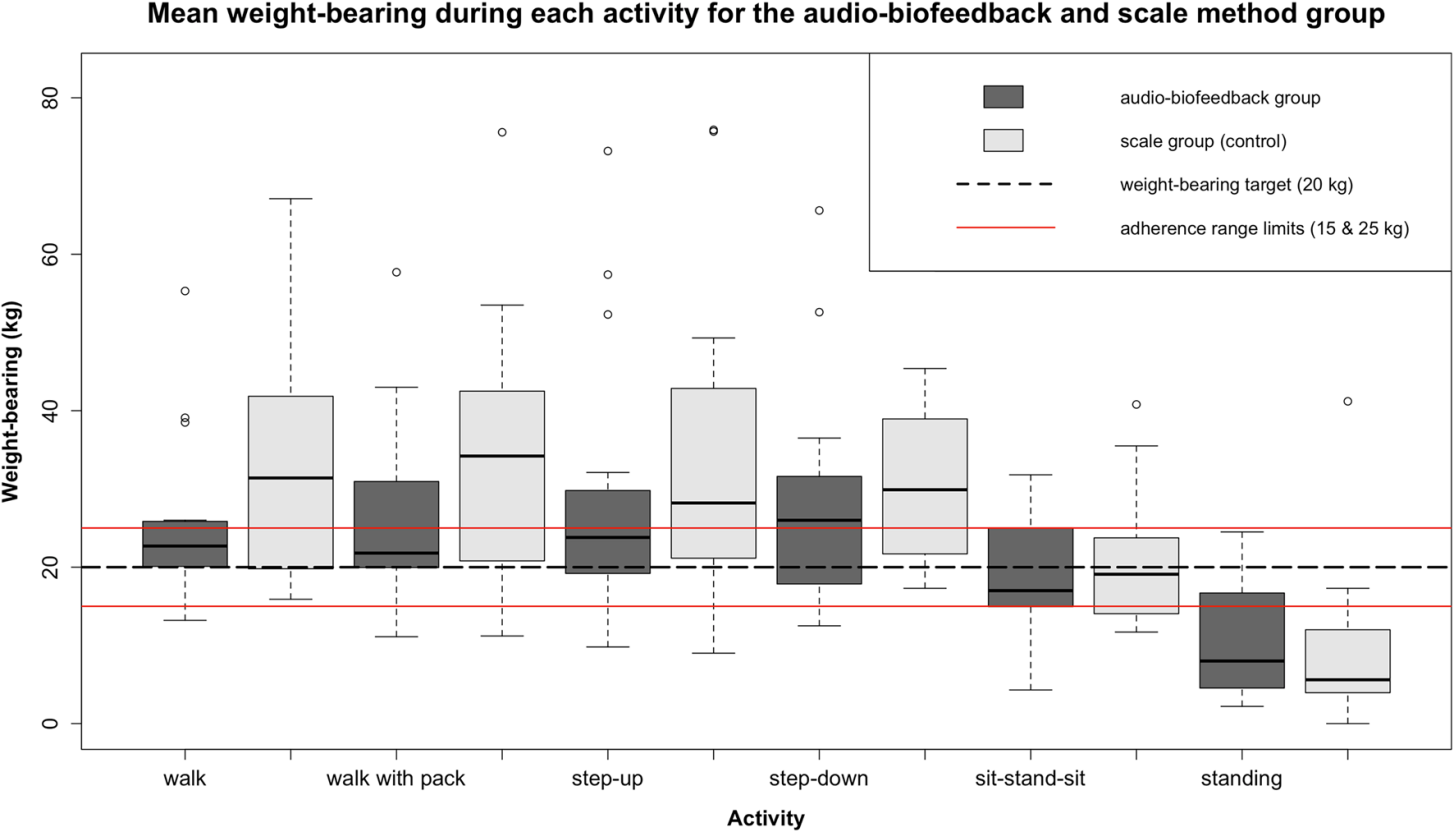
Box plots of weight-bearing for the audio-biofeedback and scale method group according to activity. This figure presents the box plots of weight-bearing (kg) of both groups for each activity against the PWB adherence zone. The 25 kg limit not to be exceeded is shown by the top horizontal line, the 15 kg lower limit by the lowest horizontal line

The primary outcome was the amount of load on the PWB leg during six functional mobility activities (Table 2). For the sit-stand-sit activity the group means of the maximum PWB load were within the adherence range of 15–25 kg. Meanwhile, the group mean load was below the 15 kg threshold for standing (10.2 ± 7.1 kg audio-feedback group versus 9.5 ± 9.9 kg control group) and over the 25 kg threshold for all walking and step activities. There were no statistically significant differences between the intervention and control groups for any of the activities measured. However, more participants in the audio-biofeedback group loaded close to or within the target range for four of the six activities than those in the scale method group (Fig. 2).

**Table 2:** Weight-bearing of the audio-biofeedback and control groups during different activities

Activity	Audio (n = 15)*	Scale (n = 15)*	95% CI	p-value
Walking^†^	25.5 ± 11.2	34.4 ± 16.4	– 19.5 to 1.7	0.130
Walking (+ pack)^†^	27.3 ± 12.2	34.3 ± 16.9	– 18 to 4	0.230
Step-up^‡^	28.8 ± 18.3	34.2 ± 20.4	– 19.9 to 9.1	0.290
Step-down^‡^	28.0 ± 14.8	35.4 ± 18.8	– 20.1 to 5.3	0.161
Sit-stand-sit^‡^	21.7 ± 16.6	22.6 ± 13	– 12.1 to 10.3	0.803
Standing^§^	10.2 ± 7.1	9.5 ± 9.9	– 5.8 to 7.2	0.575

Values are expressed as: mean ± standard deviation, or as otherwise indicated

*CI* confidence interval

*Unit: kilogram (kg)

^†^Mean of all maxima of total ground reaction force during all stance phases converted to kg

^‡^Mean of maximum ground reaction force during the activity converted to kg

^§^Mean of total ground reaction force during the activity converted to kg

On an individual level, none of the volunteers’ mean PWB load was within the 15 to 25 kg adherence range for all activities. However, some volunteers loaded within the adherence range for some activities and there is a clear trend of a better adherence rate in the intervention group compared to the control group (Table 3). For example, for the walking activity, 40% of participants in the intervention group, versus 33% in the control group, loaded within the adherence range.

**Table 3:** Percentages of adherent participants according to activity

Activity	Audio (n = 15)	Scale (n = 15)
Walking	40% (6/9)	33% (5/10)
Walking (+ pack)	47% (7/8)	20% (3/12)
Step-up	40% (6/9)	13% (2/13)
Step-down	20% (3/12)	33% (5/10)
Sit-stand-sit	47% (7/8)	47% (7/8)
Standing	33% (5/10)	7% (1/14)

Values are expressed as: percentage of participants adherent (number adherent / number non-adherent)

In an exploratory analysis, adherence was calculated on a per step basis (Table 4). The percentage of adherent steps was higher in the audio-biofeedback group than the scale method group, 41.2% and 42.8% versus 25.3% and 22.2% for walking and walking with a backpack respectively, but the differences were not statistically significant. The majority of non-adherent steps were overloading for both groups. However, the percentage of overloading steps were lower in the in the audio-biofeedback group than in the scale method group (60.5% versus 72.5% for walking, 67.4% versus 80.2% for walking with the backpack).

**Table 4:** Percentages of steps according to their load classification

Activity	Load	Audio (n = 15)	Scale (n = 15)	95% CI	p-value
Walking	Adherent	41.2%	25.3%	– 5.6 to 37.4	0.096
	Non-adherent overload	37.3%	63.5%	– 56.3 to 3.7	0.081
	Non-adherent underload	21.7%	11.2%	– 7.5 to 28.5	0.222
Walking (+ 4 kg pack)	Adherent	42.8%	22.2%	– 6.1 to 47.2	0.161
	Non-adherent overload	44.9%	64.5%	– 52.1 to 12.8	0.328
	Non-adherent underload	12.4%	13.2%	-20.0 to 18.3	0.242

Values are expressed as: mean percentage of steps, or as otherwise indicated

*CI* confidence interval

The secondary objective was to assess the influence of grip strength, MoCA score, BMI, body weight, age and sex on PWB load (kg). In the multiple linear regression analysis, three models were tested: BMI, age, and gender (model 1); BMI, age and MoCA (model 2); and BMI, age, and grip strength (model 3). Of these, model 2 had the best fit for all activities and is presented below. This model was statistically significant for four of the six activities, explaining 52% of the mean PWB load result for sit-stand-sit, 50% for the step-down, 36.5% for the step-up and 18.9% for walking (Table 5).

**Table 5:** Linear regression results for the relationship between participant characteristics and weight-bearing for different activities

Activity	Model 2*^†^	BMI^‡^	Age^‡^	MoCA^‡^	Gender^‡^	Grip ^‡^
Sit-stand-sit	52.2% (5.31e-05)	2.02 (6.251e-05)	0.29 (0.525)	– 3.70 (0.0002)	5.32 (0.329)	0.06 (0.824)
Step-down	50% (0.0001)	2.50 (1.289e-05)	0.62 (0.241	– 3.97 (0.0007)	7.13 (0.260)	0.10 (0.738)
Step-up	36.5% (0.002)	2.27 (0.001)	1.19 (0.042)	– 4.28 (0.001)	1.16 (0.872)	-0.25 (0.451)
Walking	18.9% (0.038)	1.42 (0.009)	0.68 (0.133)	– 2.37 (0.025)	3.95 (0.467)	-0.03 (0.912)

*BMI* body mass index, *MoCA* montreal cognitive assessment

*Multiple linear regression model with independent variables: BMI, MoCA, age

^†^Values expressed as percentage (p-value)

^‡^Values expressed as regression coefficient (p-value)

## Discussion

This randomised controlled trial aimed to investigate whether audio-biofeedback during PWB training could improve adherence to a 20 kg PWB limit, over the traditional scale method, in older adults. The primary hypothesis was that the intervention group would adhere to the PWB target better than the control. In this trial, the addition of audio-biofeedback did not provide a statistically significant benefit over the scale method. However, the audio-biofeedback group mean was closer to the PWB target compared to the control for all walking and step activities. Furthermore, the range in PWB means within the audio-biofeedback group was narrower than in the scale method group for most activities. This was because more individuals in the intervention group loaded within or closer to the adherence range, with fewer outliers, than in the control group. Suggesting that, although audio-biofeedback may not assure PWB adherence, it may be superior to the scale for learning loading consistency and accuracy.

Two trials with healthy participants also reported results in favour of audio-biofeedback compared to traditional instruction [12, 34]. However, being small studies without a proper control group, both are at a high risk of bias. Some authors suggest that poor adherence in studies with healthy volunteers may be because the participants are not in pain nor at risk of injury if they over-load the PWB limb. This is supported by studies that have reported a correlation between pain and underloading after traditional instruction [14, 15]. However, Dabke et al. investigated the scale method in healthy volunteers compared to patients and found that neither group was able to reproduce PWB satisfactorily [36]. Furthermore, randomised trials with patients also reported overloading during walking and no statistically significant difference between the audio-biofeedback and control group after the first training [33, 37]. Although Hershko and colleagues [37] reported the audio-biofeedback group was loading close to the PWB target after 5 days of training, Hurkmans and colleagues [33] reported poor adherence in both groups despite daily training sessions over 5 days.

Our results showed that PWB adherence varied considerably depending on the activity measured. Both groups loaded within the PWB adherence range for the sit-stand-sit, but loaded below the lower threshold for standing and above the upper threshold for the walking and step activities. This was surprising because, irrespective of the activity measured, most studies report PWB loads above targets. Although there are comparable trials for walking, no studies were found which investigated the effect of audio-biofeedback on PWB performance during the activities step-up, step-down or sit-stand-sit. In the exploratory analysis of the walking activities, the majority of steps were non-adherent for both groups and participants were more likely to load over the upper, than under the lower, threshold. Although percentages of adherent steps were higher in the audio-biofeedback group than the control group for all activities, differences were not statistically significant.

The secondary objective was to assess the influence of individual characteristics on adherence to PWB. In this trial, participants with higher BMI, lower level of cognitive function and older age were more likely to place more weight on their PWB leg. Although statistically significant, the model did not completely predict the PWB load, indicating that there are other factors involved that are not accounted for in this model. Interestingly, the strength of the relationship varied depending on the activity, suggesting that different activities may be influenced by different combinations of characteristics. The strongest correlation was between BMI and PWB load. This reflects the results of other studies [12, 17] and was expected considering all participants, regardless of weight, were instructed to load the same amount. The correlation between lower MoCA score and higher PWB load supports other literature proposing that cognitive decline may contribute to poorer PWB adherence [15]. As anticipated, older participants tended to overload more. Ageing is associated with physical and cognitive decline, which may negatively impact PWB learning and ability. Although two studies [12, 14] reported no significant relationship between older age and poorer PWB performance, age is likely a true correlator and many studies report a correlation [17, 38, 39]. Surprisingly, a trend of increased grip strength correlating with higher PWB loads, but no significant association, was shown in our study population. A prospective study investigating factors influencing PWB performance in 50 hip replacement patients also did not identify upper arm strength as a correlator [14]. It is possible grip strength may not be a significant influencing factor or that the relationship between upper body strength and the ability to PWB successfully when using crutches is more complex; for example, male gender may be a confounding factor.

Overall, our results are similar to other biofeedback studies in this population group: adherence is poor in persons over 60, regardless of training and whether healthy volunteers or patients. Because audio-biofeedback methods may provide some benefit over traditional methods, they warrant further research. In this trial, the OpenGo system was safe and easy to use, supporting its implementation in future PWB trials. However, given that scales are affordable, readily available and similarly effective, investing in such systems for clinical practice is currently not supported by research. Based on this trial and others [15, 17, 38], clinicians may want to spend time correctly training PWB using traditional methods, with feedback provided using a combination of methods, and consider influencing factors, in particular take extra care with those who are overweight, of older age, and have reduced cognitive function. Further research is needed to develop a more evidence based PWB prescription and instruction approach, one which takes both diagnosis and individual factors into account, with large-scale randomised controlled trials particularly important in the growing older adult population group.

### Trial strengths and limitations

The small sample size means a higher risk of bias and as a result, the findings of the hypothesis testing should be interpreted with caution. However, many aspects of the trial design and its execution help to reduce the risk of bias. Allocating participants through simple randomisation limits selection bias and reduces confounding and, although there was no stratification, baseline characteristics were similar between the two groups. Because healthy participants’ weight-bearing abilities may not be representative of patients, in particular those in pain, trial results cannot be generalised to the patient population. Furthermore, measurements were performed in a laboratory environment. At home or in a clinical setting without observation, persons may be more relaxed and more likely to weight-bear over or under the recommendation. These factors limit transferability to clinical practice. However, it was appropriate to investigate OpenGo audio-biofeedback in a controlled environment and in healthy older people first. Furthermore, this study is one of few that has examined auditory feedback in older adults. Not to mention the only trial, to the best of our knowledge, to investigate multiple activities besides walking.

Because there was no gold-standard protocol for the instruction of PWB, the intervention protocol was based on the methodology of peer-reviewed studies and the authors’ knowledge of clinical practice. As is typical with rehabilitation interventions, blinding of intervention allocation was not possible. A further potential source of bias is that the allocation, instruction and measurement of each and all participants was carried out by the same individual researcher. However, the allocation was concealed for all involved until the point of PWB instruction and the physiotherapist followed a detailed standardised protocol to limit instruction bias. Furthermore, any “out-of-feedback” treatment effect was minimised by the physiotherapist not being able to see results until all measurements had been completed. Measurement bias was reduced by following a measurement protocol, using valid instruments and repeated measures.

## Conclusions

This randomised trial demonstrated that, although there were differences in group means, the addition of audio-biofeedback during PWB instruction did not provide a statistically significant benefit over the scale method in healthy older adults. For both groups, means of weight-bearing of the PWB limb were adherent for the sit-stand-sit activity, but non-adherent for the standing, walking and step activities. However, audio-biofeedback was safe and, because the intervention mean was closer to the target load than the control for all walking and step activities, it may be beneficial for improving PWB accuracy. Lower MoCA score, older age and higher BMI were associated with more loading of the PWB limb. Until research is more conclusive, physiotherapists are advised to correctly employ traditional instruction, supported by feedback methods available to them, and consider contributing factors such as level of cognitive function. Further research should focus on investigating audio-biofeedback in the growing older patient population group, with multiple training sessions, and identifying thresholds at which influencing factors, such as level of cognitive function, are predictive of poorer adherence.

## Data Availability

No datasets were generated or analysed during the current study.

## Abbreviations

BMI: Body mass index
CI: Confidence interval
MoCA: Montreal cognitive assessment
PWB: Partial weight-bearing

## Footnotes

1 Jamar, Performance Health, Illinois, United States.

2 OpenGo, OpenGo, Moticon ReGo AG, Munich, Germany.

3 Hangzhou Tangsuan Network Technology Co., Ltd, China.

4 Sealed Envelope Ltd., London, United Kingdom.

5 ADE Germany GmbH, Hamburg, Germany.

6 R Core Team, Vienna, Austria.
